# Magneto-Optical Thin Films for On-Chip Monolithic Integration of Non-Reciprocal Photonic Devices

**DOI:** 10.3390/ma6115094

**Published:** 2013-11-08

**Authors:** Lei Bi, Juejun Hu, Peng Jiang, Hyun Suk Kim, Dong Hun Kim, Mehmet Cengiz Onbasli, Gerald F. Dionne, Caroline A. Ross

**Affiliations:** 1 State Key Laboratory of Electronic Thin Films and Integrated Devices, University of Electronic Science and Technology of China, No. 4 Sec. 2 Jianshe N. Street, Chengdu 610054, China; 2 Department of Materials Science & Engineering, University of Delaware, 305 DuPont Hall, Newark, DE 19716, USA; E-Mail: hujuejun@udel.edu; 3 Department of Materials Science & Engineering, Massachusetts Institute of Technology, 77 Massachusetts Ave., Cambridge, MA 02139, USA; E-Mails: peng0469@gmail.com (P.J.); hskimkim@gmail.com (H.S.K.); rogercop@mit.edu (D.H.K.); onbasli@mit.edu (M.C.O.); dionne@ll.mit.edu (G.F.D.); caross@mit.edu (C.A.R.)

**Keywords:** monolithic integration, magneto-optics, magnetic oxides, thin films, optical isolator, optical resonator

## Abstract

Achieving monolithic integration of nonreciprocal photonic devices on semiconductor substrates has been long sought by the photonics research society. One way to achieve this goal is to deposit high quality magneto-optical oxide thin films on a semiconductor substrate. In this paper, we review our recent research activity on magneto-optical oxide thin films toward the goal of monolithic integration of nonreciprocal photonic devices on silicon. We demonstrate high Faraday rotation at telecommunication wavelengths in several novel magnetooptical oxide thin films including Co substituted CeO_2−δ_, Co- or Fe-substituted SrTiO_3−δ_, as well as polycrystalline garnets on silicon. Figures of merit of 3~4 deg/dB and 21 deg/dB are achieved in epitaxial Sr(Ti_0.2_Ga_0.4_Fe_0.4_)O_3−δ_ and polycrystalline (CeY_2_)Fe_5_O_12_ films, respectively. We also demonstrate an optical isolator on silicon, based on a racetrack resonator using polycrystalline (CeY_2_)Fe_5_O_12_/silicon strip-loaded waveguides. Our work demonstrates that physical vapor deposited magneto-optical oxide thin films on silicon can achieve high Faraday rotation, low optical loss and high magneto-optical figure of merit, therefore enabling novel high-performance non-reciprocal photonic devices monolithically integrated on semiconductor substrates.

## 1. Introduction

**Figure 1:**
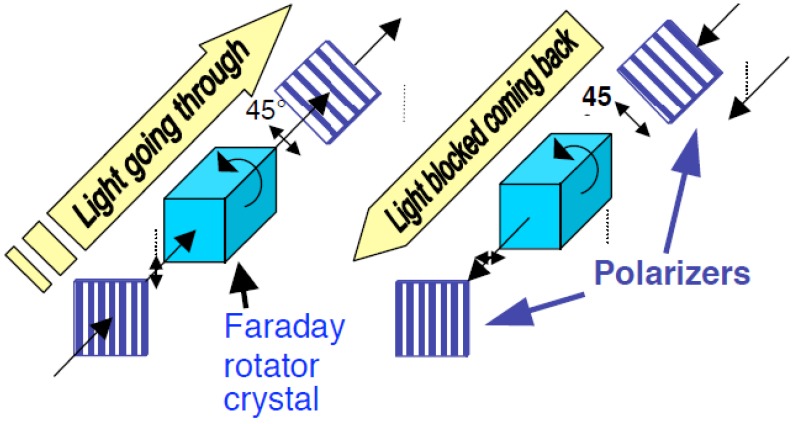
Sketch of a bulk optical isolator operating using the magneto-optical Faraday effect. The Faraday rotator is typically made from a Bi-substituted yttrium iron garnet (BiYIG) crystal.

Nonreciprocal photonic devices, including optical isolators and circulators, are indispensable components in optical communications systems. By breaking the time-reversal symmetry of light propagation, nonreciprocal photonic devices offer important functionalities, such as optical isolation and circulation in a photonic system, which is used to stabilize the laser operation or simplify the optical system design. In fiber optical systems, optical nonreciprocity is achieved by using magneto-optical (MO) materials, in which time-reversal symmetry is broken by applying a magnetic field across the MO material to achieve macroscopic spin and orbital alignment and nonreciprocal electric dipole transitions. In a discrete optical isolator device, the magnetic field is applied coaxially with the light propagation direction, which yields a nonreciprocal rotation of the polarization of linearly polarized light, namely the Faraday effect. Two 45° offset polarizers are used to construct the isolator device as shown in Figure 1, where the polarization direction of the incident light rotates 45° to pass through the analyzer. However, it rotates −45° when propagating backward and is subject to isolation by the first polarizer. The key material that enables high device performance is a MO iron garnet single crystal with both high transparency and high saturation Faraday rotation at telecommunication wavelengths (1550 nm and 1310 nm). Today, most commercial optical isolators using such materials satisfy the requirements of high isolation ratio (>20 dB), low insertion loss (<1 dB), broadband operation (several tens of nm), and superior temperature stability (−45~85 °C), which meet most lasers’ requirements in fiber optical systems.

With the development of planar lightwave circuits (PLC), optoelectronic integrated circuits (OEIC) and silicon photonics, there is an increasing demand for integrating discrete optical components on a single chip. There are several motivations for integration of such devices on a common semiconductor substrate, including cost, footprint and device reliability. While discrete optical isolators are quite costly, an integrated device can significantly reduce the cost, thereby reducing the overall budget for constructing an integrated optical system [1]. By integration, a waveguide-based optical isolator can eliminate the need for large single crystals, magnets, polarizers, reciprocal rotators and prisms, and reduce the device footprint to the sub-millimeter range. Meanwhile, an integrated optical isolator solves packaging issues including lens coupling and off-chip alignment of discrete photonic components to waveguides which are necessary for bulk isolators. These motivations make integrated nonreciprocal photonic devices highly desirable in an optical waveguide system.

In spite of the strong motivation, integrating nonreciprocal photonic devices on semiconductor substrates has been a long term challenge, and there is no practical solution for a high performance optical isolator on silicon or III-V substrates, mostly due to the material incompatibility between magneto-optical garnets and semiconductors. When integrating iron garnets on a semiconductor substrate, large lattice mismatch and thermal mismatch exist between the film and substrate. Impurity phases, film cracking and film-substrate reactions usually occur, which result in optically lossy materials and prevent the fabrication of high performance optical isolators [2]. One strategy to circumvent the thin film integration challenge is to wafer bond single crystal garnet films on a semiconductor substrate. For example, Mizumoto *et al.* demonstrated direct wafer bonding of Ce-doped Y_3_Fe_5_O_12_ (Ce:YIG) epitaxial films grown on (GdCa)_3_(GaMgZr)_5_O_12_ (SGGG) to InP, SOI and LiNbO_3_ substrates [3]. Optical isolation was demonstrated in Mach-Zehnder (MZ) and semileaky waveguide structures [4,5]. Baets *et al.* demonstrated adhesive benzocyclobutene (BCB) bonding of garnet on SOI [6,7]. Optical isolators and circulators were fabricated using MZ interferometers with high isolation ratios. Although wafer bonding is a promising method, there are disadvantages including the requirement for large substrate areas for reliable bonding [3], use of relatively high cost single crystal garnet substrates, as well as low throughput for device integration. Therefore, monolithic integration of magneto-optical oxide thin films is a preferred solution for low cost, compact size and high yield considerations, if high figure of merit *(*FoM*)* magneto-optical oxide thin films can be developed on Si or III-V substrates.

Besides the materials challenges, monolithically integrated on-chip isolators also require innovative device designs different from bulk Faraday rotators. Due to structural birefringence between TE and TM modes of an optical waveguide, the Faraday rotation can be severely degraded [8,9,10]. Novel MO device designs need to be developed to tackle these issues for device integration.

In this paper, we review our recent research activities toward monolithic on-chip integration of nonreciprocal photonic devices by developing high FoM magneto-optical oxide thin films. In Section 2, we provide a brief summary of current research status on monolithic integration of MO thin films and devices on semiconductor substrates. Then we introduce several MO oxide thin films for integration on silicon, including Co doped CeO_2−δ_, Co or Fe doped SrTiO_3−δ_ and YIG buffered CeYIG thin films in Section 3, Section 4 and Section 5 respectively. Finally, we describe a monolithically integrated optical isolator on silicon using a garnet/SOI based nonreciprocal racetrack resonator. It is worth mentioning that non-MO solutions are rapidly emerging as a promising field for integrated nonreciprocal photonics, such as nonreciprocal devices using nonlinear optics [11,12,13,14], single-sideband modulation [15] and nonreciprocal interband photonic transitions [16,17]. Given the focus of this paper on magneto-optics, these non-MO strategies will not be discussed.

## 2. Progress on Monolithic Integration of Magneto-Optical Oxides on Semiconductors

To evaluate the quality and usefulness of a magneto-optical material, a figure of merit (FoM) based on Faraday rotation is defined as:

(1) $$\text{FoM}=\frac{\Theta }{\alpha }$$

where Θ and *α* are the Faraday rotation and absorption coefficient respectively. Considering a Faraday rotator-based optical isolator, for achieving 45° Faraday rotation while maintaining less than 1 dB insertion loss, the material FoM should be larger than 45 deg/dB [18]. This is a general material selection criterion for monolithically integrated magneto-optical oxides for nonreciprocal photonic applications.

On-chip deposition of various magneto-optical oxides has been explored by different groups in the past decades. For magnetic garnet thin films, the major integration challenges are: large lattice mismatch (YIG: 12.376 Å, Si: 5.43 Å, GaAs: 5.65 Å, InP 5.87 Å), thermal mismatch (linear thermal expansion coefficient YIG: 10.4 × 10^−6^/°C, Si: 2.33 × 10^−6^/°C, GaAs: 5.73 × 10^−6^/°C) and a high fabrication thermal budget of garnet films. Various deposition methods including pulsed laser deposition (PLD) [2,19,20,21,22,23], sputtering [24,25,26,27,28,29,30,31,32,33], ion beam sputtering [34] and MOCVD [35] have been explored for YIG, Bi:YIG and Ce:YIG films. Progress, including lowering the thermal budget, increasing the phase purity, stabilizing of rare-earth substituted garnet films and reducing thermally-induced cracks, have been demonstrated in the past decade. Sung *et al.* demonstrated rapid thermal annealing (RTA) crystallization of polycrystalline YIG on silicon, which significantly reduced the crystallization thermal budget to 5 s at 750 °C [27]. By patterning the YIG waveguide before RTA crystallization, Sung *et al.* also demonstrated a crack free YIG waveguide on SiO_2_/Si substrate [31]. Köner *et al.* demonstrated stabilization of Bi_3_Fe_5_O_12_ growth by a 100 nm YIG seed layer on SiO_2_, with a strong Faraday rotation observed in the bi-layer film stack [17]. Meanwhile, nonreciprocal photonic devices based on polycrystalline garnet materials have been demonstrated on semiconductor substrates, including waveguide Faraday rotators [32], quasi phase matched magneto-optical waveguides [36,37,38,39] and magneto-optical photonic crystals [40,41,42,43,44,45,46,47]. Novel devices including unidirectional Bloch oscillators [48], photonic crystal magneto-optical circulators [49,50,51,52] and nonreciprocal optical resonators [53,54,55] have also been proposed. In spite of this progress, the optical loss of doped YIG films has not been systematically studied. Polycrystalline garnet films with high enough FoM on silicon, and novel waveguide-based nonreciprocal photonic devices such as optical isolators on a semiconductor substrate, are still challenging goals. 

Exploring other silicon or III-V compatible magneto-optical oxides with high FoM is another route taken by researchers. Spinels, including CoFe_2_O_4_, γ-Fe_2_O_3_ and MgFe_2_O_4_, *etc.* show better lattice match with silicon. Epitaxial growth of MgAl_2_O_4_ on Si has been demonstrated using MOCVD, suggesting its integrability with silicon [56]. However, the performance-limiting factor is the strong absorption of these materials caused by the intra-atomic electric dipole transition, intervalence charge transfer (IVCT) and intersublattice charge transfer (ISCT) mechanisms [57]. Tepper *et al.* successfully integrated epitaxial γ-Fe_2_O_3_ on MgO substrates using pulsed laser deposition [58,59,60,61]. The film showed both high Faraday rotation of 2.5°/μm and high absorption loss of 3.5 × 10^5^ dB/cm at 1550 nm wavelength. The high absorption was attributed to the high concentration of Fe^2+^ ions. Suzuki *et al.* deposited Zn and Al doped CoFe_2_O_4_ films on fused quartz substrate [62]. The magneto-optical FoM was characterized in the visible to near infrared wavelength range, with the highest FoM observed to be ~0.6 deg/dB at 1550 nm, which is mostly limited by the Co^2+^ absorption. In MgFe_2_O_4_, although Fe is mostly in the 3+ valence state, the ISCT mechanism can cause optical absorption as high as ~600 cm^−1^ at around 1550 nm wavelength [61,63]. Reducing the material absorption is therefore highly desired to increase the magneto-optical FoM.

One way to reduce the absorption loss in the spinel materials is to use their nanocrystals. Motivated by the discovery of about one order of magnitude higher optical transparency in γ-Fe_2_O_3_ nanocrystals compared to their bulk counterparts [64], nanocrystal spinel ferrite composites [65,66] and optical waveguides [67,68] have been developed by various researchers and characterized in the near infrared wavelength range. Guerrero *et al.* demonstrated a Verdet constant of 0.23°/G·cm in γ-Fe_2_O_3_/SiO_2_ nanocomposites [66]. Choueikani *et al.* integrated a magneto-optical waveguide on silicon using CoFe_2_O_4_ nanoparticles embedded in a SiO_2_/ZrO_2_ matrix [67]. Faraday rotation of 310°/cm and a FoM of 2.3°/dB was observed at 1550 nm wavelength, which is higher than that of a CoFe_2_O_4_ thin film or bulk materials. However, the reduced oscillator strength of the electric dipole transition also caused lower Faraday rotation of these materials. Despite improved performance, the relatively low Faraday rotation, superparamagnetic behavior, and low FoM still need to be addressed for practical nonreciprocal device applications.

Other room temperature ferromagnetic materials include manganites ((La,Sr)MnO_3_, (La,Ca)MnO_3_, *etc.*) and orthoferrites (LaFeO_3_, BiFeO_3_ *etc.*). For manganites, the metallicity caused by Mn^3+^–O^2−^–Mn^4+^ double exchange results in strong absorption and limits the magneto-optical FoM [69]. For orthoferrites, thin films usually show low magnetization [70], multi-orientation of crystals [71] and strong birefringence [72], therefore limiting their application in integrated magneto-optical devices.

Table 1 summarizes several magneto-optical oxides’ saturation Faraday rotation, extinction coefficient, FoM, and their challenges for application in on-chip nonreciprocal photonic devices. In general, different challenges need to be overcome for different oxides to achieve a FoM sufficiently high for device application. Novel magneto-optical thin film materials are therefore highly desirable to resolve these challenges. In the following sections, we will review our recent research on developing high FoM magneto-optical oxide thin films toward monolithic integration of nonreciprocal photonic devices on silicon.

**Table 1:** Magneto-optical properties of several magnetic oxides and challenges for applications in on-chip nonreciprocal photonic devices.

Magnetic Oxides	Θ (°/cm) at 1550 nm	α (dB/cm) at 1550 nm	FoM(°/dB) at 1550 nm	Challenges
*Garnets* (Y_3_Fe_5_O_12_, (Bi,Y)_3_Fe_5_O_12_, (Ce,Y)_3_Fe_5_O_12_)	−3300 (CeYIG) [73]	5.8 [73]	570	Lattice and thermal mismatch; Impurity phases; Thermal budget
*Spinel/Hexaferrites* (Fe_3_O_4_, γ-Fe_2_O_3_, CoFe_2_O_4_, MgFe_2_O_4_, BaFe_12_O_19_)	2.5 × 10^4^ (γ-Fe_2_O_3_) [58]	3.5 × 10^5^ [60]	0.07	Absorption due to Fe^2+^, Co^2^^+^, IVCT, ISCT *etc.*
*Manganites* ((La,Sr)MnO_3_, (La,Ca)MnO_3_)	~2000 (LSMO at 2.6eV) [69]	~8 × 10^4^ [69]	–	Metallic and optically absorbent due to Mn^3+^–Mn^4+^ double exchange
*Orthoferrites* (YFeO_3_, LaFeO_3_, BiFeO_3_)	~500 [74]	<2 [75]	250	Low magnetization in thin films; Birefringence; Thermal budget
*Nanocrystals* (γ-Fe_2_O_3_, CoFe_2_O_4_ nanocrystals)	310 [67]	130 [67]	2.4	Relatively low Faraday rotation, superparamagnetism, relatively low FoM

## 3. Co Substituted CeO_2−δ_ Films

**Figure 2:**
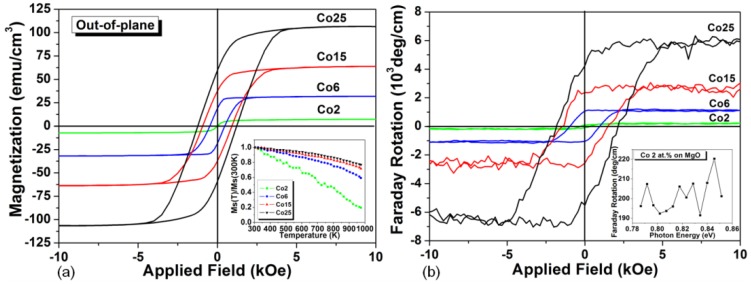
(**a**) Room temperature out-of-plane magnetic hysteresis of Co-substituted CeO_2−δ_ films, with Co2, Co6, Co15 and Co25 standing for 2 atom %, 6 atom %, 15 atom % and 25 atom % Co substituted CeO_2−δ_ respectively. The inset shows the temperature dependence of saturation magnetization (*M*_s_) normalized to *M*_s_ at 300 K; (**b**) Room temperature out-of-plane magneto-optical hysteresis of Co doped CeO_2−δ_ films. The inset shows the saturation magnetization as a function of incident photon energy for the Co2 sample [80]. (Reprinted with permission from [80]. Copyright 2008 AIP Publishing LLC).

One of the most closely lattice matched oxide with silicon is CeO_2_, with a fluorite lattice structure and a cubic lattice constant of 5.411Å. CeO_2_ shows only 0.35% lattice mismatch with silicon along the (100) crystal orientation, therefore it can be epitaxially grown on either (100) or (111) oriented silicon substrates. CeO_2_ also shows a low optical absorption in the visible to near infrared wavelength range [76]. Room temperature ferromagnetic properties of Co or Fe doped CeO_2_ have been reported by a number of groups [77,78,79]. However, their magneto-optical properties have rarely been studied. Using PLD, we deposited room temperature ferromagnetic Co doped CeO_2−δ_ films on MgO and SrTiO_3_ substrates up to 25 atom % Co substitution [80]. The film thicknesses range from 260 nm to 950 nm, with less cracking observed in the thinner films. The films showed increasing saturation magnetization and coercivity with Co substitution, with an out-of-plane magnetic anisotropy as shown in Figure 2a. Strong room temperature Faraday rotation from −200 deg/cm up to −6900 deg/cm at 1550 nm wavelength has been observed in these films, as shown in Figure 2b, which is among the highest Faraday rotations at this wavelength range [81,82,83,84]. No obvious magneto-optical dispersion was observed in the near infrared wavelength range. As the Co content increased, absorption increased and FoM decreased. A FoM of 0.07 deg/dB was obtained in 2 atom % Co doped CeO_2−δ_ films.

The ferromagnetic properties of the films were discussed in terms of a magnetoelastic spin alignment as observed in other dilute magnetic oxide systems [85]. The linear dependence of magnetization *versus* temperature of the Co2 film was also observed in other DMS systems [85]. Co^4+^ ions (low-spin) may be responsible for the high temperature magnetism and magneto-optical properties. The splitting of the *t*_2*g*_ triplet provides the magnetoelastic stabilization to extend the linear *M*_s_-*T* slope to high temperatures, while the spin-orbit interaction also satisfies the selection rule and provides high temperature Faraday rotation [86]. Meanwhile, Ce^3+^ may also provide strong electronic dipole transitions in the near infrared wavelength range, which may be partly responsible for the large negative Faraday rotation. However, with increasing Co concentration, the optical absorption quickly increases and results in decreased magneto-optical FoM. The strong absorption may be caused by defect bands [87] or impurity phases when Co concentration is high [88]. The contributions of multiple valence state Co ions and Ce ions to electric dipole transitions create uncertainty about the source of the Faraday rotation and magnetic coercivity, which could only be unraveled by further studying the material microstructure and cation valence states.

## 4. Fe or Co Substituted SrTiO_3−δ_ Films

Another material showing good silicon compatibility is SrTiO_3_. Excellent optical transparency has been demonstrated in SrTiO_3_ thin films at communication wavelengths [89]. Moreover, by adopting a 45° rotation growth mode [90], it can also be epitaxially grown on silicon (100) substrates. Room temperature ferromagnetism has been observed in transition metal ion doped perovskites such as Co doped (La,Sr)TiO_3_ [91,92,93] and Fe doped BaTiO_3_ [94]. The high solubility of transition metal ions on the Ti site, as well as the tunability of larger ions on the Sr site qualify SrTiO_3_ as an ideal starting point for developing new MO materials for nonreciprocal photonic applications.

**Figure 3:**
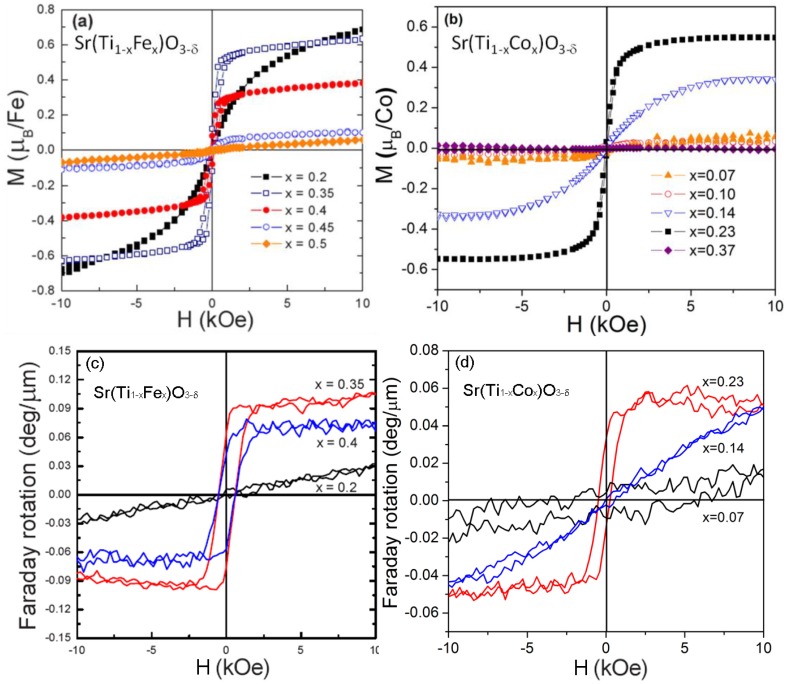
Room temperature out-of-plane magnetization hysteresis of (**a**) STF and (**b**) STC films with different Fe or Co concentrations. Also shown is the RT out-of-plane Faraday rotation hysteresis at 1550 nm wavelength for (**c**) STF and (**d**) STC respectively [95,96]. (Reprinted with permission from [95,96]. Copyright 2010 IOP Publishing Ltd and Deutsche Physikalische Gesellschaft).

Fe or Co substituted SrTiO_3−δ_ thin films (namely STF and STC respectively) were deposited by PLD with thickness ranging from 200 nm to 300 nm on SrTiO_3_, La_0.3_Sr_1.7_AlTaO_6_ (LSAT) and CeO_2_/YSZ buffered silicon substrates. No obvious cracks were observed in all films [95,96,97]. For thin films on all substrates, structural analysis, including XRD, TEM, XPS and XANES on STF and STC films, showed no impurity phases [95,96]. The lattice constants of STF and STC films increased monotonically with transition metal ion doping, which is a reverse trend compared to their bulk counterpart. This is a result of the oxygen deficiency in the film and the resulting lower valence state and larger radius of Fe and Co ions. Both STF and STC films grown on SrTiO_3_ substrates showed in-plane compressive strain and a tetragonal unit cell with c axis oriented along the out-of-plane direction. With increasing Fe or Co concentrations, a paramagnetic to ferromagnetic to antiferromagnetic transition was observed. Figure 3a,b shows the room temperature M-H hysteresis of Fe or Co substituted SrTiO_3−δ_ films. For thin films on LSAT substrates, saturation moments of 0.6 μ_B_/Fe and 0.5 μ_B_/Co were observed in Sr(Ti_0.65_Fe_0.35_)O_3_ (STF35) and Sr(Ti_0.77_Co_0.23_)O_3_ (STC23) films respectively which suggest some antiferromagnetic coupling. Such films also showed a strong out-of-plane anisotropy, with anisotropy field up to 6000 Oe at room temperature and no preferential magnetization direction in-plane. Based on the magnetic moment and magneto-elastic effects, the populations of different valence state Fe ions were calculated as Sr^2+^[${\text{Ti}}_{0.65}^{4+}{\text{Fe}}_{0.1}^{4+}{\text{Fe}}_{0.25}^{3+}$]O_2.88_ for a sample of STF35 [97]. Similar magnetic moments and magnetic anisotropy was observed in films on silicon. A saturation Faraday rotation of −780 deg/cm and −500 deg/cm at 1550 nm wavelength was demonstrated in STF35 and STC23 films on LSAT as shown in Figure 3c,d. Different from garnet films, the magneto-optical hysteresis of these materials showed an out-of-plane anisotropy (like the magnetic hysteresis), which may be beneficial for developing TE mode nonreciprocal phase shift devices, where the magneto-optical thin film needs to be magnetized out-of-plane [98]. The optical constants of STF40 and STC23 films were characterized by ellipsometry. At 1550 nm, for STF40 and STC23 the optical constants were *n* = 2.2 and *k* = 2 × 10^−3^ and *n* = 2.34 and *k* = 1.1 × 10^−3^. The FoM of STF40 and STC23 were therefore determined to be 1.1 deg/dB and 0.57 deg/dB respectively in this experiment.

**Figure 4:**
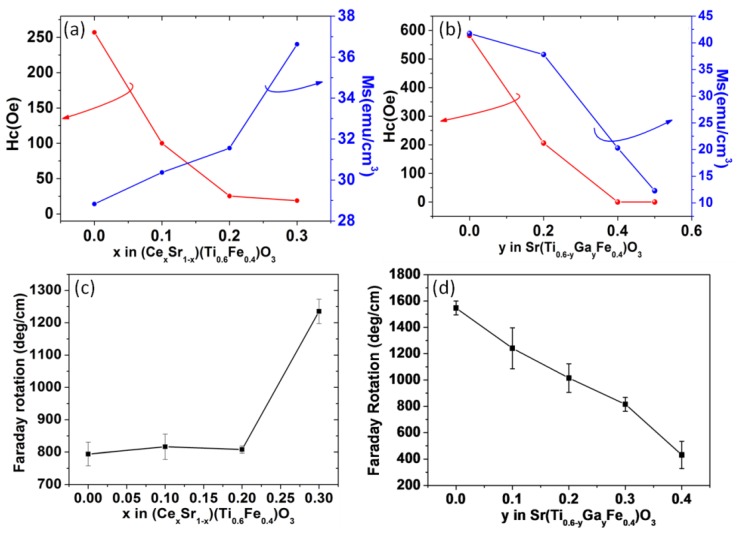
Room temperature saturation magnetization and coercivity of (**a**) Ce substituted STF and (**b**) Ga substituted STF films on LSAT substrates. Also shown are the Faraday rotations as a function of dopant concentration for (**c**) Ce substituted STF and (**d**) Ga substituted STF films [99,100] on LSAT. (Reprinted with permission from [99,100]. Copyright 2011 AIP Publishing LLC).

**Figure 5:**
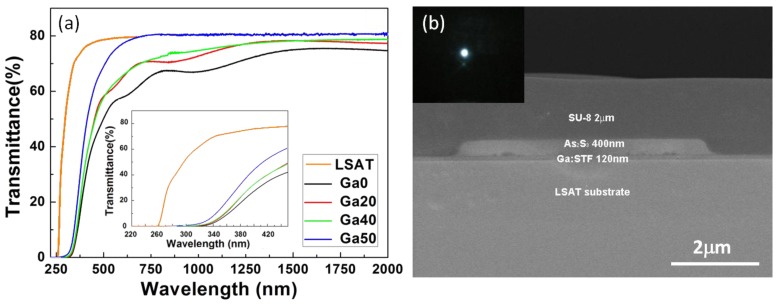
(**a**) Optical transmittance spectra of Ga:STF films with the inset showing a close-up view of the optical band gap region; (**b**) Cross-sectional SEM image of an As_2_S_3_/Ga:STF strip-loaded waveguide with an SU-8 top-cladding layer fabricated on an LSAT(001) substrate. The inset shows the guided mode profile at 1550 nm wavelength [99]. (Reprinted with permission from [99]. Copyright 2011 AIP Publishing LLC).

To further improve the material FoM, donor or acceptor doping in the STF material was carried out by partially substituting Sr^2+^ with Ce^4+^ and Ti^4+^ with Ga^3+^ respectively. For films grown on silicon or LSAT substrates, a systematic increase or decrease of the saturation magnetization and Faraday rotation was observed upon Ce^4+^ or Ga^3+^ substitution respectively as shown in Figure 4, indicating the strong dependence of the material magnetization on cation valence states. On the other hand, the optical transparency followed a reverse trend compared to the Faraday rotation, indicating a trade-off between Faraday rotation and optical transparency. This trade-off can be mitigated by choosing proper dopants and concentrations; therefore, a higher magneto-optical FoM can be achieved [99,100]. Figure 5 shows the optical transparency of Ga-doped STF films and a strip-loaded waveguide fabricated on an LSAT substrate. The thin film optical loss was estimated by simulating the modal profile and confinement factor. For Sr(Ti_0.2_Ga_0.4_Fe_0.4_)O_3−δ_, the material showed −400 deg/cm Faraday rotation and 100~120 dB/cm optical loss at 1550 nm, suggesting a FoM of 3~4 dB/cm. Further improvement of the material FoM may be achieved by enhancing Faraday rotation by Bi or Ce doping on the Sr site, or better control of the Fe valence states to lower the optical loss.

**Figure 6:**
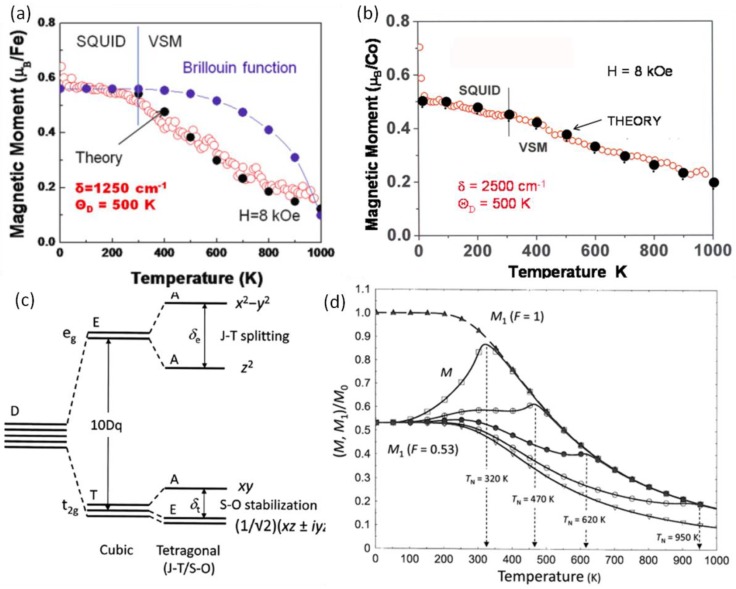
*M*_s_-*T* curve of (**a**) STF35 and (**b**) STC23 films and the theoretical fitting using Equation (2) or the Brillouin function in (**a**); (**c**) The crystal-field energy-level diagram of a 3d ion in an oxygen octahedron subject to in-plane biaxial compressive stress producing a tetragonal (Jahn-Teller type) distortion, where 10 Dq, δ*_t_* and δ*_e_* stand for the crystal field splitting energy between *e_g_* and *t*_2*g*_ orbitals, the splitting energy of *t*_2g_ orbitals due to spin-orbital coupling (S-O stabilization), and the splitting energy of *e_g_* orbitals due to Jahn-Teller effect (J-T stabilization); (**d**) Computed *M*-*T* curves for a series of Neel temperatures with *F* = 0.53 using the generic model described by Equation (3), where *M*_1_, *F*, *T*_N_ stands for moment of “isolated” ions, population of “isolated” ions and the Neel temperature respectively [96,97,101]. (Reprinted with permission from [96,97,101]).

The origins of ferromagnetic and magneto-optical properties in these films were further studied. The saturation magnetization *M*_s_ of the STF and STC films showed a linear dependence with temperature, as shown in Figure 6a,b, which is very different from a conventional exchange coupled ferromagnetic system, where the *M*_s_-*T* curve follows the Brillouin function showing a characteristic nonlinear convex contour. These observations suggest that exchange coupling is not the dominant mechanism for the observed ferromagnetism. Compared to dilute magnetic semiconductor (DMS) systems [85], a similar *M*_s_-*T* dependence was observed, which is attributed to the effects of lattice strain. Unlike a DMS, when the cation concentrations are high, exchange coupling, for example double exchange of Fe^3+^–O^2−^–Fe^4+^ and superexchange Fe^3+^–O^2−^–Fe^3+^, becomes important. This introduces more spin alignment mechanisms [95], making the system more complicated. However, when Fe and Co concentrations are substantially increased, antiferromagnetic superexchange dominates, and both films became antiferromagnetic. 

A generic model considering strain and superexchange coupling provides a more comprehensive understanding of the magnetic behavior in these films. The ferromagnetism is believed to originate from magnetoelastic spin ordering [85]. For a transition metal ion in an oxygen octahedron under biaxial compressive stress and *z*-axis expansion, the 3d orbital crystal-field energy-level diagram is shown in Figure 6c. Considering a single ion subject to a pure Jahn-Teller singlet stabilization, the occupation probability of electrons in the upper and lower *e_g_* orbitals follows the Boltzmann distribution. Therefore, the magnetoelastic effect on the saturation magnetization can be described by:

(2) $$\frac{M_{s}(T)}{M_{s}(0)}\approx \text{tanh}[\frac{\delta_{JT}(0)\text{tanh}(\Theta_{D}/2T)}{2kT}]$$

where the Jahn-Teller splitting δ*_JT_*(*T*) of the *e_g_* states in the first tanh function is decreased by the onset of vibronic modes of the oxygen ligand coordination as *T* approaches the Debye temperature Θ*_D_* and is expressed in terms of a band-narrowing second tanh function. When the magnetic ion concentration increases, the general model for magnetization temperature dependence can be described as follows based on the presence of antiferromagnetic superexchange in the oxide where at least two ions are neighbors [101]:

(3) $$\frac{M}{M_{0}}=\text{tanh}[\frac{\delta_{JT}(0)\cdot \text{tanh}(\Theta_{D}/2T)}{2kT}]\times \{F+(1-F)[1-B_{S}(a)]\}$$

where *F* and (1−*F*) describes the populations of “isolated” and “exchange coupled” ions in the lattice, and *B_S_*(*a*) is the temperature-dependent Brillouin function. The resulting *M*/*M*_0_ *vs.* *T* curves are shown in Figure 6d. Using this model, the *M*_s_-*T* curves are well fitted to experimental measurement results of STF and STC films as shown in Figure 6a,b. 

For magneto-optical properties, the electric dipole transitions contributing to the MO properties need to follow the selection rules. Although several ions such as Co^2+^ or Fe^2+^ may contribute to the magnetization following the magnetoelastic spin ordering scenario, only Fe^4+^ and Co^4+^ in low-spin state can satisfy the selection rules. Therefore they are the only possible contributors to the Faraday rotation [86]. This finding indicates that, by driving the ions toward a higher valence state (while maintaining the material’s magnetization), one may further improve the FoM. Further study on the MO spectroscopy of these films may provide more experimental insights to improve these oxide thin films.

## 5. Polycrystalline BiYIG and CeYIG Films

As we discussed in Section 2, integration of magnetic garnet thin films on semiconductor substrates has been widely studied for monolithically integrated nonreciprocal photonic devices because garnets have excellent FoM. Despite this, an integrated magnetic garnet film with high Faraday rotation and high FoM remains a challenge. To achieve this goal, we introduced a thin (~20 nm) crystalline YIG seed layer for BiYIG and CeYIG growth. The YIG layer not only stabilized the garnet phase of BiYIG and CeYIG, but also significantly decreased the thermal budget of BiYIG or CeYIG growth. By using a rapid thermal anneal process to crystallize the YIG seed layer [102], the overall thermal budget for the garnet film stack can be significantly reduced, which is beneficial to reduce cracking in the garnet films and degradation of other parts of the device.

**Figure 7:**
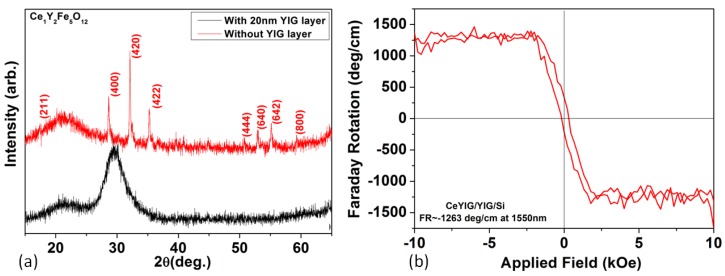
(**a**) XRD spectrum of CeYIG films deposited on SiO_2_/Si substrates with and without the YIG seed layer; (**b**) Room temperature out-of-plane Faraday rotation spectrum of a 500 nm thick polycrystalline CeYIG film deposited on silicon [102]. (Reprinted with permission from [102]. Copyright 2009 Society of Photo Optical Instrumentation Engineers).

High phase purity doped garnet thin films can be deposited using this method. Figure 7a shows the XRD comparison of 80 nm (Ce_1_Y_2_)Fe_5_O_12_ film (CeYIG) deposited with or without a 20 nm thick crystallized YIG seed layer on a SiO_2_/Si substrate using PLD. These film thicknesses are chosen to reduce thermal mismatch induced cracks. For the same deposition condition, the CeYIG on the YIG seed layer crystallized as pure garnet phase without other phases according to X-ray diffraction, while a control sample without YIG was amorphous. The CeYIG film had a lattice constant of 12.46 Å, very close to to that of a single crystal [102]. Thanks to the lower processing temperature and film thickness, the thermal stress between the CeYIG film and Si substrate remained below the threshold for thermal crack generation, and the film showed a low surface RMS roughness of 0.92 nm with few cracks. A similar process was also applicable to Bi_1.8_Y_1.2_Fe_5_O_12_ films (Bi1.8YIG). The Bi1.8YIG/YIG and CeYIG/YIG bilayer films showed room temperature saturation magnetizations of 125 emu/cm^3^ and 120 emu/cm^3^ respectively, suggesting that 91 vol % and 87 vol % of the films were crystallized into the garnet phase. Room temperature saturation Faraday rotation at 1550 nm wavelength was −838 deg/cm for Bi1.8YIG and −830 deg/cm for CeYIG, compared to over −3000 deg/cm and −3300 deg/cm reported for sputter-deposited epitaxial films on garnet substrates. As a comparison, the polycrystalline YIG only showed +160 deg/cm Faraday rotation at 1550 nm. The lower Faraday rotation in both BiYIG and CeYIG films may result from incomplete incorporation of Bi or Ce ions into the lattice, or off-stoichiometry leading to rare earth or Fe ions with unwanted valence states. The latter assumption is supported by observation of a higher Faraday rotation in CeYIG films upon decreasing the oxygen partial pressure during deposition. A Faraday rotation of −1263 deg/cm was achieved in CeYIG films deposited in an oxygen pressure of 5 mTorr, as shown in Figure 6b.

**Figure 8:**
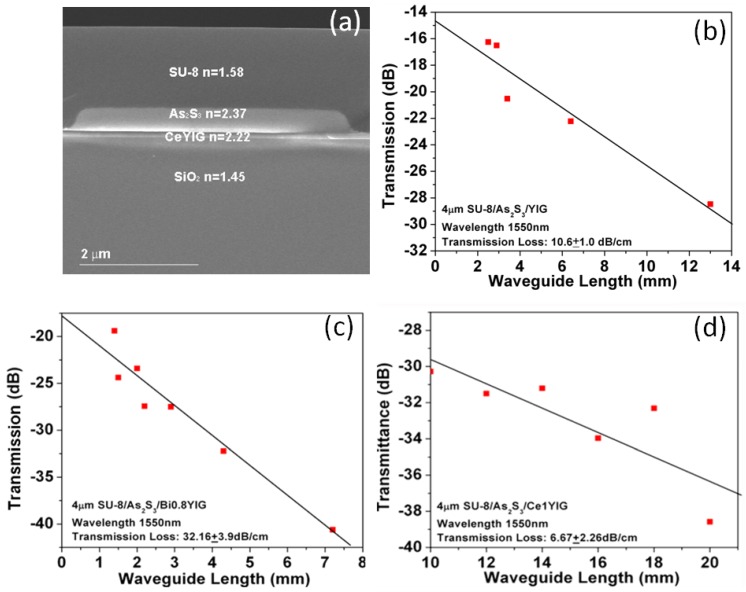
(**a**) Cross-sectional SEM image of a 4 μm wide As_2_S_3_/CeYIG strip-loaded waveguide. Also shown are the waveguide transmission loss determined using cut-back method for; (**b**) As_2_S_3_/YIG; (**c**) As_2_S_3_/Bi0.8YIG; and (**d**) As_2_S_3_/CeYIG films [102]. (Reprinted with permission from [102]. Copyright 2009 Society of Photo Optical Instrumentation Engineers).

Another important concern is the optical loss of the polycrystalline garnet films. Due to the small film thickness and low optical absorption, spectroscopy or ellipsometry methods are insufficiently accurate for loss characterization. To resolve this issue, we used a chalcogenide glass (ChG)/garnet thin film strip-loaded waveguide for evaluation of absorption [102]. The ChG materials are highly transparent in the near infrared wavelength range. Waveguides based on ChGs were formed by a simple lift-off process for fast prototyping [103]. A cross-section SEM image of such a waveguide is shown in Figure 8a. Using the cut-back and “paperclip” methods [102], the total waveguide loss can be accurately measured as shown in Figure 8b–d. By comparing the loss value with a baseline waveguide loss of pure ChG strip waveguides, as well as the simulated confinement factor in the CeYIG layer, the thin garnet film loss could be determined. Another advantage of using this method is to prevent etching and patterning-induced loss in the garnet thin films, therefore capturing the material-related intrinsic loss of the films. From these measurements, we demonstrated that losses of YIG, Bi0.8YIG and CeYIG films were around 50 dB/cm, 150 dB/cm and 40 dB/cm respectively. The loss from Bi1.8YIG was too high to perform a valid cut-back or “paperclip” measurement, which is possibly due to a tendency for bismuth segregation at polycrystal grain boundaries [104]. From both the Faraday rotation measurement and the loss characterization, the *FoM* of polycrystalline YIG and CeYIG films were determined as 2 deg/dB and 21 deg/dB respectively.

## 6. A Nonreciprocal Ring Resonator Based Optical Isolator on SOI

**Figure 9:**
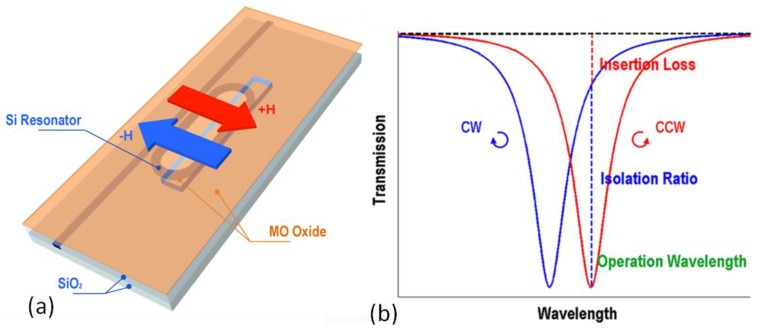
(**a**) Sketch of a patterned nonreciprocal optical resonator; (**b**) Transmission spectrum of clockwise (CW, forward) and counter-clockwise (CCW, backward) propagating light through the resonator device. Optical isolation is achieved around the CCW resonance wavelength [105]. (Reprinted with permission from [105]. Copyright 2011 Nature publishing group, Macmillan Publishers Limited).

The demonstration of high quality magneto-optical films enables monolithic optical isolator integration. In conventional device designs, the device footprint is still large due to the weak magneto-optical effect. Considering a CeYIG film with −830 deg/cm Faraday rotation, a Mach-Zehnder interferometer and Faraday rotator optical isolator will require device lengths of 5 mm and 542 μm, respectively. To reduce the device footprint, resonator device structures have been proposed including ring resonators [53,54,55] and photonic crystals [49,50,51,52]. We demonstrated a patterned nonreciprocal optical racetrack resonator which allowed uniaxial magnetic field operation, maintained a high device FoM and had a compact device footprint [105]. The device structure is shown in Figure 9a. A silicon racetrack resonator was coated with an oxide cladding layer in which a window was opened and a magneto-optical film was deposited, so that only part of the resonator was covered by the magneto-optical thin film. When applying an in-plane magnetic field perpendicular to the light propagation direction in the resonator, a nonreciprocal phase shift (NRPS) was produced in the patterned resonator region, therefore allowing non-degenerate resonance frequency splitting with respect to forward and backward propagating light, as shown in Figure 9b. Optical isolation is achieved around the backward propagation resonance wavelength, as indicated by the dashed line in Figure 9b.

The nonreciprocal resonance shift of this device can be analytically derived based on resonator optical theory [105]. If we define the garnet-clad and silica-clad resonator section lengths to be *L*_1_ and *L*_0_ respectively, the resonance wavelength degeneracy ∆γ can be expressed as:

(4) $$\Delta \lambda =\frac{FSR\cdot L_{1}\Delta \beta_{TM}}{2\pi },\text{ where }FSR=\frac{{\lambda_{r}}^{2}}{L_{0}n_{g0}+L_{1}n_{g1}}$$

where FSR is the free spectral range of the resonance wavelength, *n_g_*_0_, *n_g_*_1_ are the group index of the silica-clad and garnet-clad waveguide sections respectively, and ∆*β_TM_* stands for the NRPS of the garnet-clad waveguide section. The loaded cavity quality factor of the resonator can be derived as:

(5) $$Q=\frac{\pi (L_{0}n_{g0}+L_{1}n_{g1})}{\lambda_{r}\alpha L}$$

where *α* stands for the averaged optical absorption per length of the whole racetrack resonator. 

The patterned resonator design maintains a high device FoM. For a resonator based optical isolator, we define the device figure of merit as:

(6) $$F_{res.}=\frac{2\Delta \lambda }{w}=\frac{\Delta \lambda }{\lambda_{r}}\cdot Q_{in}\approx \frac{L_{1}\Delta \beta_{TM}}{\alpha L}(\text{near}\;\text{critical}\;\text{coupling})$$

where the intrinsic quality factor *Q* ≈ 2*Q* near critical coupling. When the optical loss of the silica-clad waveguide region is comparatively low, and the major loss is from the garnet-clad waveguide section, we obtain:

(7) $$F_{res.}=\frac{L_{1}\Delta \beta_{TM}}{\alpha_{1}L_{1}}=\frac{\Delta \beta_{TM}}{\alpha_{1}}$$

This result states that the isolator device FoM is only determined by the magneto-optical waveguide NRPS and optical loss. Therefore the performance of this device directly follows the FoM of the material.

**Figure 10:**
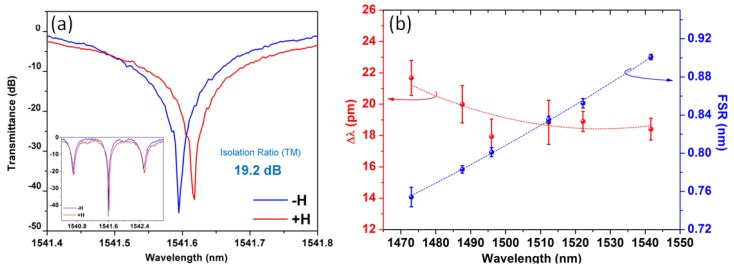
(**a**) TM-mode transmittance spectra as a function of the applied magnetic field at near critical coupling resonance; (**b**) Wavelength dependence of the nonreciprocal resonance peak shift and the *FSR* [105]. (Reprinted with permission from [105]. Copyright 2011 Nature publishing group, Macmillan Publishers Limited).

The isolation performance of TM polarized light near 1541 nm wavelength is shown in Figure 10a. By end-coupling of the infrared laser light through the resonator device with an applied magnetic field up to ±1500 Oe, nonreciprocal resonance shift was observed. The isolation ratio, insertion loss and 10 dB operation bandwidth of the prototype device were 19.5 ± 2.9 dB, 18.8 ± 1.1 dB and 1.6 GHz respectively. Wavelength dependences for both the nonreciprocal resonance shift and the FSR have been measured at different resonance wavelengths, as shown in Figure 10b. The relatively low dispersion of ∆*λ* is due to a compensation between negative magneto-optical dispersion of the CeYIG material and the positive dispersion of the device FSR. The relatively high insertion loss for the current device is mostly due to fabrication issues, such as etch-induced loss and garnet thin film covering on the waveguide sidewalls. For insertion loss reduction and broad band operation, future work needs to improve both the integration process and the magneto-optical material, as well as to develop broad band device structures with a compact device footprint. Meanwhile, temperature stability also needs to be resolved by material and device design to meet practical device application requirements.

## 7. Summary

In this paper, we have reviewed our recent research on several magneto-optical thin film materials toward the goal of monolithic integration of nonreciprocal photonic devices on silicon. Novel silicon-compatible magneto-optical oxides, including Co-substituted CeO_2−δ_, Co/Fe-substituted SrTiO_3−δ_ and YIG buffered CeYIG thin films show properties of high Faraday rotation, perpendicular magnetic anisotropy or high magneto-optical FoM at communication wavelengths. Therefore, they are promising candidates for device integration. We have summarized these materials’ Faraday rotation, optical absorption and FoM in Table 2. The Ga doped STF and polycrystalline CeYIG films show high FoM approaching the requirement of 45 deg/dB. Recently, a higher FoM of 56 deg/dB has been reported in sputter-deposited polycrystalline CeYIG films [106]. This progress has significantly improved the FoM of magneto-optical thin films on semiconductor substrates, and paved the way for device integration.

**Table 2:** Magneto-optical thin film candidates for on-chip nonreciprocal photonic devices at 1550 nm discussed in this paper.

Materials	FR (deg/cm)	Loss (dB/cm)	FoM (deg/dB)
Ce_0.94_Co_0.06_O_3−δ_ [80]	−200	~3000	0.06
Sr(Ti_0.77_Co_0.23_)O_3−δ_ [96]	−200~−500	300~400	0.57
Sr(Ti_0.6_Fe_0.4_)O_3−δ_ [95]	−780	700	1.1
Sr(Ti_0.2_Ga_0.4_Fe_0.4_)O_3−δ_ [99]	−400	100~120	3~4
Y_3_Fe_5_O_12_ (polycrystal) [102]	+100	~50	~2
Ce_1_Y_2_Fe_5_O_12_ (polycrystal) [102]	−830	~40	~21

On the device side, we have demonstrated an integrated optical isolator on silicon using a nonreciprocal optical resonator. To the best of our knowledge, this is the first monolithically integrated optical isolator on silicon. We compare this device to nonreciprocal photonic devices using garnet epitaxial films on single crystal garnet substrates, as shown in Table 3. 

**Table 3:** Comparison of the resonator based optical isolator on silicon to integrated isolators on garnet using epitaxial thin films.

Devices	Isolation Ratio (dB)	Insertion Loss (dB)	Device Length (μ)
NRMC (Nonreciprocal Mode Conversion) [107]	27	8~11	4100
NRMC [108]	24	4.6	3150
NRMC [109]	13.3	6.3~8.1	4500
MZ NRPS [110]	19	13	8000
MZ NRPS [111]	21	8	4000
MZ NRPS [112,113]	28	11(2.2 dB exess loss compared to waveguide with CeYIG)	1500
MZ NRPS (BCB bonding) [6]	25	14	960
Resonator NRPS [105]	19.5	18.2	290

Our current device has shown advantages of compact device size, uniaxial magnetic field operation and controllable magneto-optical dispersion. However, it also has disadvantages of small isolation bandwidth, high temperature sensitivity and the requirement of a (unidirectional) magnetic field. Also, the fabrication induced device loss needs to be reduced. For future developments, novel devices with compact footprint, broad operation bandwidth and wide temperature range stability need to be developed. With the development of monolithically integrated magneto-optical thin films and novel device designs, we are confident that we will see the fulfillment of the goal of monolithic integration of nonreciprocal photonic devices on semiconductor substrates in the near future.
